# Uthando Lwethu (‘our love’): a protocol for a couples-based intervention to increase testing for HIV: a randomized controlled trial in rural KwaZulu-Natal, South Africa

**DOI:** 10.1186/1745-6215-15-64

**Published:** 2014-02-20

**Authors:** Lynae A Darbes, Heidi van Rooyen, Victoria Hosegood, Thulani Ngubane, Mallory O Johnson, Katherine Fritz, Nuala McGrath

**Affiliations:** 1 Center for AIDS Prevention Studies, University of California, 50 Beale Street, San Francisco, CA 94105, USA; 2 Human Sciences Research Council, Sweetwaters, Mbubu Road, Sweetwaters, South Africa; 3 University of Southampton, University Road, Southampton SO17 1BJ, UK; 4 Centre for Health and Population Studies, University of KwaZulu-Natal, Durban, South Africa; 5 International Center for Research on Women, 1120 20th Street NW, Suite 500 North, Washington, DC, USA

**Keywords:** Couples-based HIV testing, CHCT, Couples-based intervention, HIV prevention, South Africa

## Abstract

**Background:**

Couples-based HIV counseling and testing (CHCT) is a proven strategy to reduce the risk of HIV transmission between partners, but uptake of CHCT is low. We describe the study design of a randomized controlled trial (RCT) aimed to increase participation in CHCT and reduce sexual risk behavior for HIV among heterosexual couples in rural KwaZulu-Natal, South Africa. We hypothesize that the rate of participation in CHCT will be higher and sexual risk behavior will be lower in the intervention group as compared to the control.

**Methods/design:**

Heterosexual couples (N = 350 couples, 700 individuals) are being recruited to participate in a randomized trial of a couples-based intervention comprising two group sessions (one mixed gender, one single gender) and four couples’ counseling sessions. Couples must have been in a relationship together for at least 6 months. Quantitative assessments are conducted via mobile phones by gender-matched interviewers at baseline, 3, 6, and 9 months post-randomization. Intervention content is aimed to improve relationship dynamics, and includes communication skills and setting goals regarding CHCT.

**Discussion:**

The Uthando Lwethu (‘our love’) intervention is the first couples-based intervention to have CHCT as its outcome. We are also targeting reductions in unprotected sex. CHCT necessitates the testing and mutual disclosure of both partners, conditions that are essential for improving subsequent outcomes such as disclosure of HIV status, sexual risk reduction, and improving treatment outcomes. Thus, improving rates of CHCT has the potential to improve health outcomes for heterosexual couples in a rural area of South Africa that is highly impacted by HIV. The results of our ongoing clinical trial will provide much needed information regarding whether a relationship-focused approach is effective in increasing rates of participation in CHCT. Our intervention represents an attempt to move away from individual-level conceptualizations, to a more integrated approach for HIV prevention.

**Trial registration:**

Study Name: Couples in Context: An RCT of a Couples-based HIV Prevention Intervention

ClinicalTrials.gov identifier: NCT01953133.

South African clinical trial registration number: DOH-27-0212-3937

## Background

The estimated prevalence of HIV in South Africa is 17.9% among 15- to 49-year-olds and 39.5% among female antenatal clinic attendees in KwaZulu-Natal [1], indicating a continuing need for effective HIV prevention. Further, recent studies in sub-Saharan Africa found 60% to 94% of new HIV infections occur within marriage or co-habiting heterosexual partnerships [2]. South Africa has low rates of marriage compared to many other sub-Saharan African countries, and also has a high level of partner non-cohabitation in non-marital and marital couples, often due to migration by both partners [3]. Recent national HIV testing campaigns in South Africa have improved rates of individual testing [4], but in rural KwaZulu-Natal the percent of men knowing their HIV status (55%) still lags behind women (83%) [5]. Project Accept, a community-level randomized controlled trial (RCT), found high rates of multiple partnerships (32%) among men aged 18 to 32 years in rural South Africa [6]. In contrast, longitudinal population-based data from another area of rural KwaZulu-Natal suggests that the proportion of men having multiple or concurrent sexual partnerships has been declining in recent years, to approximately 12% and 6% of men aged 17 to 54 years reporting multiple and concurrent sexual partners, respectively, in 2011 [7]. Other challenges concerning HIV status disclosure also remain. For example, in a cohort of HIV-positive individuals in rural KwaZulu-Natal, many do not know their partner’s HIV status and do not disclose their own positive status [8]. Since 1992, couples-based HIV counseling and testing (CHCT) is a proven strategy to reduce the risk of HIV transmission between partners due to mutual disclosure of HIV status [9]. However, uptake of CHCT is low [10-12]. Reasons include: a lack of environments that are welcoming to couples and men; the need for both partners to be willing to participate, which could be impacted by the presence of intimate partner violence, or unwillingness to disclose HIV serostatus; and a lack of community mobilization and support for CHCT [10]. These circumstances signal the need for HIV prevention interventions that: 1) target couples; 2) increase uptake of CHCT; and 3) examine the role of relationship factors on testing and sexual behaviors.

Several benefits of CHCT were identified by the World Health Organization (WHO) working group on CHCT [13]. First, CHCT provides a springboard for early antiretroviral therapy (ART) initiation, which has been found to be highly efficacious for reducing morbidity and mortality and in reducing HIV transmission within serodiscordant partnerships [14]. Second, CHCT provides a foundation for both primary and secondary HIV prevention. For example, recent evidence points towards dramatic decreases in sexual risk following knowledge of serodiscordance via CHCT. Rosenberg and colleagues [15] reported that unprotected intercourse decreased from 71% to 8% in 1 month following knowledge of serodiscordance via CHCT among couples who participated in the Partners in Prevention HSV/HIV Transmission Study in three sites in South Africa. The authors suggested that it is the awareness of their discordant serostatus as a couple which is the driver of the reduction in sexual risk, as opposed to an individual level of knowledge of HIV-positive serostatus. CHCT could also impact sexual risk behavior with outside partners, thereby reducing HIV transmission to secondary partners. Third, CHCT has the potential to improve the success of safer conception strategies, an approach that is enhanced by the increasing availability of pre-exposure prophylaxis (PReP) and early ART initiation. Similarly, CHCT has the potential to improve uptake, retention, and adherence to family planning, prevention of mother-to-child transmission (PMTCT), and ART-focused interventions. Finally, the WHO also identified increased emotional support and positive relationship outcomes as potential benefits for couples that participate in CHCT. However, even given these potential benefits, no prior intervention study has had CHCT as its outcome.

Prime among its benefits is that CHCT requires mutual disclosure. Given this aspect of the procedure, consistent findings of effectiveness of CHCT in reducing sexual risk are not surprising. This mutual knowledge provides a solid foundation from which to make mutual decisions about sexual behavior with each other, and provides an opening for discussions regarding HIV. However, couples must be psychologically ready to enter into the testing process together [16], which can be more threatening than finding out one’s own status. Testing as a couple can result in knowledge that could threaten a relationship, and escalate fear of results in terms of one’s own health, one’s partner’s health, and the stability of a relationship [17]. From a relationship-based perspective, it is understandable why rates for CHCT are low; avoiding activities that may threaten a relationship may be protective of relationships.

CHCT is a highly effective strategy towards preventing new HIV infections; but motivations to participate in couples-based testing are relationship-focused, which can complicate the decision-making process. Partners want to establish trust, demonstrate commitment to each other, and know their HIV status prior to embarking on future decisions such as having children together, or getting married [18]. Evidence from our research group [19] and others [20-26] indicates that a comprehensive approach that includes such components as education and skills-building in couples’ communication is necessary to successfully tackle the myriad of issues facing heterosexual couples in sub-Saharan Africa.

While the benefits of CHCT are clear, gaps remain in our knowledge regarding couples and HIV prevention. When examining prior investigations of couples-based interventions, several issues emerge. First, couples-based interventions have typically adapted individually-focused interventions for couples [27,28]. Second, most couples-based HIV prevention interventions have included only couples where both partners know their own and their partner’s HIV status when evaluating sexual risk behavior outcomes [29]. However, men often use their female partner’s status as a proxy for their own HIV status, without ever having been tested themselves [30]. Often CHCT is, indeed, the intervention itself [9,31,32], and the goals of the intervention have been to determine the effectiveness of participating in CHCT as a risk reduction strategy. Third, other sexual behavior risk factors are important to consider, including the presence and frequency of unprotected sex with both primary and outside concurrent partners [33,34]. Finally, relationship factors have been shown to be associated with condom use, such that longer-term relationships often result in low or inconsistent condom use [35-37]. For example, Vamos and colleagues found, from their couples-based intervention in Zambia, that couples who were more positive about their relationship engaged in significantly higher condom use. These findings indicate that interventions that improve and enhance couples’ relationship dynamics may also have potential to exert change on behavioral outcomes that can improve HIV prevention efforts [38].

Prior couples-based approaches to HIV prevention have employed individually-focused theoretical orientations, thereby neglecting the influence that partners play in decisions around HIV, both its prevention and potential transmission. Interdependence theory [39,40] provides a framework for understanding relationship dynamics and emotional responses in the context of intimate relationships. It has been applied in studies of couples to explore how relationship dynamics (for example, commitment) influence relationship outcomes such as longevity, divorce, and infidelity [41,42]. Lewis and colleagues [43] extended the interdependence theory to examine both the initiation and maintenance of health-enhancing behaviors. Their model proposes that engaging in health-enhancing behaviors is the end result of relationship dynamics, such as relationship satisfaction and commitment, which are essential predisposing factors for a ‘transformation of motivation’. This transformation produces a shift in partners from an individually-centered perspective to one that is more relationship-oriented. Once this shift occurred, a couple then engages in joint efforts and cooperative action to achieve or maintain positive outcomes. For example, both partners would choose to engage in less HIV risk with outside partners in order to lessen the chances of HIV transmission for both of them, as opposed to only one partner choosing to do so.

The Prevention and Relationship Enhancement Program (PREP) is one of the most studied couples-based relationship education interventions, aimed at improving relationship success. It has demonstrated long-term (2- and 5-year) positive effects on relationship outcomes (for example, relationship satisfaction) [44]. It is based on behavioral marital therapy (BMT [45]) that focuses on problem-solving skills for couples and communication skills. Long-term outcomes have also included lower levels of relationship conflict [44]. The areas focused on in PREP are closely aligned with the relationship factors emphasized in interdependence theory [46] (improving intimacy, commitment, and so on). In addition, PREP includes identification of specific relationship goals. Our intervention approach is therefore based upon a combination of the theoretical perspective of interdependence theory and empirical findings; we hypothesize that couples that have more intimacy, stronger commitment, and clearer communication about sexuality and HIV are more likely to test for HIV together.

In sum, understanding and intervening within the dyadic context of HIV risk in South Africa is a critical component of reducing new HIV infections. Further, prior interventions have not examined predictors of participating in couples-based testing, and most couples-based interventions have only included serodiscordant couples [9,47]. The Uthando Lwethu study represents an effort to address the gaps in our knowledge by testing the efficacy of a couples-based behavioral intervention in increasing uptake of CHCT and reducing sexual risk behavior for HIV among heterosexual couples living in rural KwaZulu-Natal, South Africa. As knowledge of status is a gateway to HIV treatment and sexual risk reduction opportunities, increasing testing for HIV within partnerships is a high-impact outcome.

## Methods/design

### Objectives

Uthando Lwethu is a couples-based RCT to test the efficacy of providing relationship-based counseling (for example, targeting communication skills, improving commitment, and intimacy) to increase the rates of CHCT among a population of heterosexual couples in rural KwaZulu-Natal, South Africa. A second primary outcome is reduction of sexual risk behavior (that is, condom use with primary partner in the last 3 months). We hypothesize that by improving positive relationship dynamics among couples, their rates of CHCT will increase and the frequency of sexual risk will decrease.

### Design

**Figure 1:**
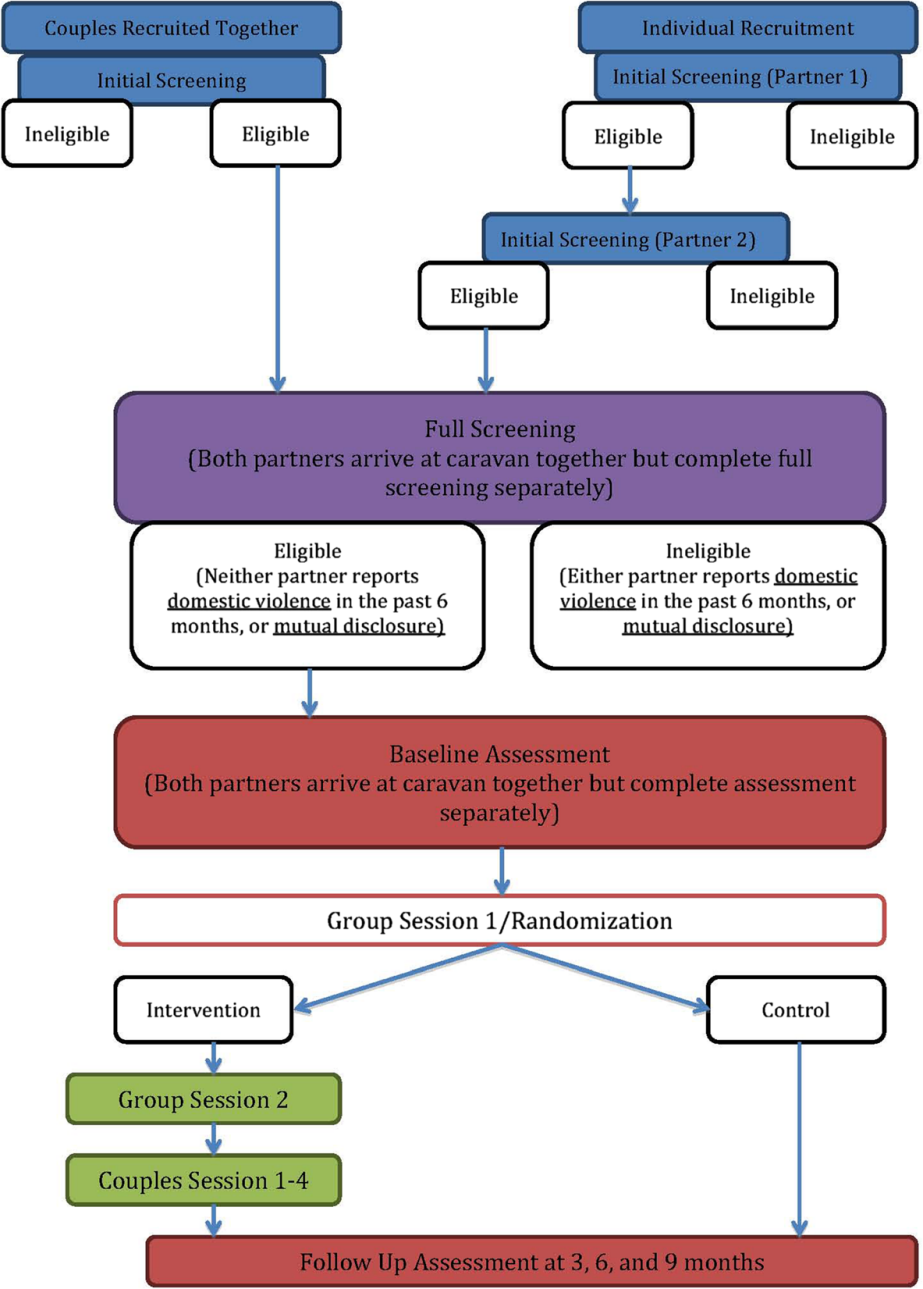
Recruitment flow chart.

This is a RCT. We aim to recruit a baseline sample of 350 heterosexual couples, which will be evenly distributed between the intervention and control groups (see Figure 1 for flow diagram). The intervention is delivered to groups of approximately 20 couples per block. At the end of the first group session, facilitators randomize couples to one of two groups. Random allocation within blocks was used to ensure adequate balance for the subsequent blocks. Couples randomized to the intervention group are scheduled for the second group session, and couples in the control group are scheduled for their 3-month follow-up assessment. Couples randomized to the control group receive no additional intervention activities, and couples randomized to the intervention group receive one additional group session and four couples’ counseling sessions. Follow-up surveys are completed for both groups at 3, 6, and 9 months post-randomization.

The intervention design of the control group participating in the first group session was chosen for several reasons. First, given the innovative approach, which aims to promote couples-based testing, there is no ‘standard of care’ that can be provided to the control group. All participants (control group and intervention), therefore, receive a group-based intervention, which provides information and education pertaining to health-related issues relevant to the community, including HIV.

### Study population

Our goal is to obtain a sample of couples from the community of interest who are appropriate for our relationship-focused, couples-based intervention. As such, we chose to include couples: whose ages are between 18 to 50 years (focusing on reproductive age), who have been in a relationship with an opposite sex partner for 6 months or longer (to ensure they have developed sufficient time together to potentially benefit from the couples’ counseling sessions), who are not in a polygamous marriage (our intervention was developed for primary partnerships, and not enough information is known about how these dynamics may differ for those in polygamous relationships), and who are sexually active (as one focus of the intervention is reduction of sexual risk behavior). Because a goal of the intervention is to encourage couples to participate in CHCT, couples are ineligible if they have previously tested for HIV together. Similarly, couples are ineligible if both partners have disclosed their HIV status, as this suggests they already possess the skills necessary to communicate about HIV with their partners and have taken the steps to know their HIV status. Our intervention, due to its focus on communication skills and enhancing positive relationship dynamics, is not appropriate for couples who are experiencing intimate partner violence. However, given the high rates of intimate partner violence in this context, couples are not excluded for reporting a history of violence within the relationship, as this would limit our ability to generalize to other couples in the community.

### Setting

The study is being conducted in Vulindlela, a rural sub-district in the province of KwaZulu-Natal, South Africa. Located approximately 150 km north-west of Durban, the sub-district has a population of approximately 500,000. Vulindlela is characterized by high unemployment as illustrated by the provincial unemployment rate of 39%. Per capita income is low with up to 30% of the households in the sub-district reporting an annual income of less than USD $1,200 [48,49].

HIV prevalence in KwaZulu-Natal was estimated to be 15.3% among 15- to 24-year-olds and 23.5% among ≥25-year-olds in 2008 [50]. Between 2004 and 2011, HIV incidence in a similar rural district of KwaZulu-Natal was estimated to be 2.63 new infections per 100 person-years [51].

### Human subjects’ protection and trial oversight

Ethical approval has been obtained from The Committee on Human Research of the University of California, San Francisco, CA, USA, and the Research Ethics Committee of the Human Sciences Research Council, Pretoria, South Africa. The study has a Data Safety and Monitoring Board (DSMB) comprising behavioral scientists and a statistician who will monitor study progress. Initial and ongoing meetings with a Community Advisory Board (CAB) ensure that the study is responsive to the needs of the study community, and that the content and procedures are culturally appropriate.

### Research activities and procedures

#### Pilot activities and trial preparation

Overall, the development of the intervention content for the pilot and the trial was informed by our collective experience in couples-based research, including the influence of relationship dynamics on sexual behavior [52], adherence to ART [53], and HIV testing [54]. Prior experience investigating issues such as recruitment of couples for research in this geographical context [55] and factors impacting couples’ relationships in this context [3,56], and Project Accept (HPTN 043), a large community-level randomized trial which was conducted at our site [57,58], also informed our approach. The instruments and intervention content were derived through prior work with heterosexual couples in South Africa and through adaptation of content from prior interventions, such as PREP. Adaptation for the communities of interest was guided by conducted formative work via qualitative interviews and focus groups in Soweto, South Africa [18,54], including community members with experience of couples’ issues (religious leaders, community leaders, health care staff). The content was later edited to reflect the cultural environment of rural KwaZulu-Natal via feedback from the study’s CAB and study staff members. We also conducted investigations regarding feasibility and acceptability for a couples-based relationship-focused intervention via interviews with couples and key informants [18].

We incorporated lessons learned from our pilot work into the intervention approach. For example, we conduct our group sessions on the weekends in order to accommodate both partners’ schedules. We developed visual aides to guide the administration of measurements that utilize Likert response scales, as prior experience found that participants often did not utilize the whole range of the response choices.

#### Study procedures

##### Recruitment and assessment

Participants are recruited through the community by a staff of trained recruiters using active and passive recruitment strategies [55]. Active strategies include approaching couples in areas such as markets, taxi ranks, and community events. Male and female recruiters are used, as our prior experience has demonstrated that same-gender recruiters often yield better results, and couples are more open to being approached by a mixed-gender team of recruiters. Recruiters also engage with community-based organizations. Recruitment flyers and posters are placed at community-based agencies and other venues. Most recruitment activities are conducted via the use of a mobile caravan (divided into partitions that allow for privacy), which allows study staff to conduct activities across a large area. The staff members use the caravan as a base for activities such as screening participants as well as conducting baseline interviews.

After providing verbal consent, each partner is screened for initial eligibility separately via a brief assessment, either in person or by telephone, and told that eligibility cannot be determined until their partner is also screened. Specifically, we assess age, relationship length, whether the couple is sexually active, and polygamous marriage. If both partners are eligible based on these criteria, a secondary screening is conducted. This is done immediately following the initial screening, if the couple is able and willing to do so, or is scheduled for a later time. The second screening requires written informed consent and assesses the full inclusion criteria, as described above. If, at the completion of the secondary screening interview, both partners remain eligible for participation, they are invited to enroll in the trial, and if they agree, they are asked to complete a baseline survey.

The baseline survey takes about 60 minutes and data are gathered by the study staff using mobile phones using a specified survey software package (Mobenzi Researcher, KwaZulu-Natal, South Africa; http://www.mobenzi.com/researcher/home[59]). The survey covers demographics, relationship characteristics, relationship dynamics (for example, satisfaction, communication), alcohol use, HIV testing history, disclosure of HIV status and HIV testing, attitudes about CHCT, sexual behavior with primary and secondary partner(s), intimate partner violence, gender-based power, and whether the couple has participated in CHCT.

#### Financial remuneration

Participants are provided with modest remuneration for their participation, primarily to compensate for travel costs. A reimbursement of 30 rand (approximately USD $3, as of January 2014) is provided for the secondary screening. Each partner is reimbursed 50 rand (approximately USD $5, as of January 2014) for participation in the group sessions. For the assessments, the participants are provided with 80 rand (Approximately USD $7, as of January 2014) for the baseline assessment, and 50 rand for each follow-up assessment.

### Uthando Lwethu intervention

The Uthando Lwethu intervention comprises six sessions (two group and four couples’ counseling sessions). All enrolled couples participate in the first group session prior to randomization, which couples attend together, and range in size from approximately 12 to 20 couples per session (approximately 4 hours). The first session is co-led by a team of male and female facilitators. For the second session (only including the couples randomized to the intervention), couples arrive together, but are split into separate men’s and women’s groups (approximately 4 hours) led by a gender-matched facilitator. The final four sessions are 90-minute couples’ counseling sessions, in which the couples meet with a counselor. Additional details on the sessions are provided below, as well as being summarized in Table 1.

**Table 1:** Description of intervention components

**Intervention component**	**Attendees**	**Component description**	**Themes in the session**	**Content of session**/**key points covered**
Group session 1	All couples prior to randomization	1. Half-day workshop	Tuberculosis (TB), including information on:	TB symptoms and spread
		2. Co-led by a male and female facilitator		
				What to do if exposed to TB
		3. Information/education		
		4. Randomization at the end		How one can get tested for TB
				TB treatment
			Alcohol	Overview of alcohol consumption
				Discussion of the prevalence of alcohol use in the community
				How alcohol use affects community members
				Information regarding the size of a ‘standard’ drink
				Risks of increased alcohol consumption
				Benefits of drinking less alcohol
			Sexually transmitted infections (STIs)	Factors that can increase exposure to STIs
				Treatment and prevention of STIs
				General information (for example, that some STIs have no symptoms in women)
			HIV/AIDS	How HIV is transmitted
				Behaviors that put one at risk for HIV
				Information about HIV testing (including the window period)
				Basic information about CHCT
			Reproductive health	Female menstrual cycle
				Family planning
Group session 2	Intervention for couples only, split by gender	1. Half-day workshop	General discussion on relationship dynamics	Group discusses relationship dynamics (for example, commitment, trust, intimacy, satisfaction)
		2. Co-led by a male and female facilitator		
		3. Information/education		
		4. Randomization at the end		How they define these concepts
			Sexual risk reduction	Potential risk for HIV for partners in long-term relationships
				Female and male condom demonstration and practice
				Guidelines on safer sex negotiation, HIV/STIs, and gender
				CHCT overview
				HIV treatment (including PMTCT and treatment as prevention)
			Skills practice	Condom demonstrations (male and female)
			Community attitudes about gender and relationships	Group discussions of pictures depicting possible scenarios in their community and how they would respond (for example, a woman getting harassed by men, a couple purchasing condoms)
				Gender-based power (raise awareness of the potential for and consequences of gender-based power differentials in relationships)
				Concurrent partnerships (including possible gender differences in attitude about concurrent partnerships, and community attitudes about them)
			Speaker-Listener technique	Speaker-Listener technique is a component of the PREP couples’ relationship education program; it is aimed to improve communication between partners and improve listening skills and empathy for one’s partner [60].
			Theories covered in group session 2: interdependence theory (role of relationship dynamics), the theory of gender, and power (discussion of gender roles and experiences)	
			PREP adaptation: aspects included from the PREP relationship education approach include communication skills and identifying relationship goals	
Couples’ counseling sessions	Intervention for couples only	1. Four couples’ counseling sessions	Session 1: introduction and goal setting	Overview of couples’ counseling sessions; explanation of couples’ skills training; discussion of relationship expectations; identification of goals for counseling; communication skills
		2. 90 to 120 minutes each		
		3. Based on PREP		
			Session 2: communication skills and identification of goals pertaining to CHCT	Identification of barriers/facilitators to meeting that goal, revisit communication skills; guided identification of communication enhancement goals; practice of communication skills; problem-solving skills; identifying goals; identification of community support
				This session also includes two exercises that encourage couples to think about actions that their partner does that they may not always take notice of or appreciate
				‘Forgetting something good’ (all original items translated from Zulu from the PREP program, other context-relevant items were added to the list based on staff feedback). The list was adapted to make it culturally appropriate for the rural South African context, and include such things as parenting activities or spending time with their partner
				‘Words of the heart’ asks each partner to quickly circle positive words that come to mind when they think of their partner (for example, ‘supportive’, ‘respect’, ‘fun’) (all words were translated into Zulu from the PREP program materials). They share the list with each other, which encourages feelings of intimacy and can serve as a reminder of the positive aspects of their relationship and their partner.

			Session 3: review goals pertaining to CHCT	Identifying positive aspects of partner and relationship; review priority issues around HIV; review communication and problem-solving skills; review goals and plans for change
			Session 4: managing change and future goals	Review of sessions, goal setting and changes, identifying future goals, discussion of maintenance of change; discussion of possible future events that could impact relationships (for example, children, HIV testing, changes in HIV status, health status changes), identifying future actions to demonstrate love/commitment to partner; closure

CHCT, couples-based HIV counseling and testing; PMTCT, prevention of mother-to-child transmission; PREP, Prevention and Relationship Enhancement Program; STI, sexually transmitted infection; TB, tuberculosis.

#### First group session

The first group session is an information session focused on health topics, including tuberculosis (TB), alcohol use and abuse, sexually transmitted infections (STIs), HIV/AIDS (including HIV testing and CHCT), risk reduction, and reproductive health (including family planning). HIV/AIDS is not the sole focus of this session, but key information points are presented, and participants engage in interactive group discussion on these topics. For each topic, key information points are discussed, and any misunderstandings or common misperceptions are corrected by the facilitator. Participants are provided with handouts containing information on community resources for topics covered (for example, health clinics, testing clinics, and so on). Finally, the couples are given an overview of the intervention, and the randomization procedure that occurs at the conclusion of this session.

#### Second group session

The second intervention session involves intervention couples only (approximately 6 to 10 couples), and occurs approximately 1 week following the first group session. The groups are conducted separately but simultaneously for the men and women and are led by gender-matched facilitators, as the topics covered in this session are of a more sensitive nature. This session focuses on relationship dynamics and how they impact HIV risk. All of the topics include a reference to HIV/AIDS. Communication skills are introduced in this session via the introduction of the Speaker-Listener technique, which is a component of the PREP couples’ relationship education program designed to improve communication between partners and improve listening skills and empathy for one’s partner [60]. It is introduced in the group session and practiced with other men or women, as opposed to one’s partner, in order for the partners to have experience with it prior to their first couples’ counseling session.

#### Third group session

The third session is the first couples’ counseling session. An overview of the four couples’ counseling sessions is given, and the couples are asked to discuss their thoughts and reactions to the two group sessions with their counselor. Informed by interdependence theory, this session focuses on strategies to both improve relationship dynamics and increase couples’ motivation to engage in mutually beneficial behavior such as CHCT. During the session, the couple is invited to discuss their expectations about their partner and their relationship, and how these expectations impact their attitudes towards HIV testing or other issues pertaining to HIV risk or transmission. They re-visit the Speaker-Listener technique (with the guidance to initially pick a relatively benign issue), and are given an introduction to problem-solving skills training adapted from the PREP curriculum. The couples are asked to practice skills learned prior to the next couples’ session.

#### Fourth group session

The fourth session/second couples session continues to focus on communication and relationship skills-building. The couples engage in a discussion about communication, and their perception of strengths and weaknesses in their communication with each other. The couple revisits their expectations around HIV testing and discusses barriers and facilitators to meeting their goals with regard to HIV testing (or other HIV-relevant goals). They select their priority goal, and then practice skills such as problem solving and the Speaker-Listener exercise to focus on how to remove barriers to meeting their goals. Their goals are revisited and reviewed, and new goals are set up, if necessary. The couple is also asked to think of different types of support in their community that they can rely on for both their relationship and their goals pertaining to HIV testing (for example, friends or family who they can talk to or receive tangible support from such as child care or transportation).

#### Fifth group session

The fifth session/third couples session is a mirror of the previous session. Following a brief check-in, the couple either continues to work on the goal from the previous session, if a satisfactory resolution has not been obtained, or focuses on a second goal that they identify for themselves with regard to HIV testing or other aspects of HIV (for example, sexual risk reduction or treatment). They continue to practice communication and problem-solving skills to come to a mutually satisfactory resolution or decision in regard to their goals for themselves as a couple. In addition, the couple engages in two exercises adapted from the PREP program aimed to help them remember or identify positive aspects of their relationship (see Table 1). The couple sets a goal for the upcoming week.

#### Final session

The final session offers a chance for the couple to review their progress in meeting their goals. No new skills or topics are introduced; rather, the counselor reviews their progress from the first session through to the final one. They discuss whether they met their goals with regard to any decisions regarding HIV testing or other HIV-related issues. The couple then discusses any challenging upcoming events that may be in their future and discuss strategies to use the skills they have used to meet them. They discuss the resources that they have identified in their community and the skills that they have learned. The final exercise is an exploration of their commitment to each other. The counselor assists them with identifying actions they can do for their partner to demonstrate their love and commitment to each other. Couples receive a certificate of completion.

All four couples’ counseling sessions have to be completed within 90 days post-randomization.

#### Control group description

In the first group session (prior to randomization), all participants are given pamphlets with information about community resources for testing for HIV as individuals and couples, including the testing resources provided by the project. All couples that have been enrolled to the study (intervention and control group) receive text messages twice a month reminding them about the availability of the service. These reminders are for both the intervention and control groups. Control couples are able to receive a condensed version of the couples’ counseling sessions following the completion of their 9-month follow-up interview, should they desire it. They are reminded at their final follow-up session (9-months post-randomization) that they can now receive these sessions. If they indicate their interest by contacting the study team, they receive two couples’ counseling sessions, which focus on providing communication skills (for example, Speaker-Listener technique) and a discussion of an issue pertaining to HIV which they identify. This condensed version is offered so that the control couples benefit from trial participation, but timed to not adversely impact between-group comparison.

#### Retention

Enrolling both partners increases the challenge of retention, and we have implemented several strategies. First, each participant is asked to provide a primary and secondary contact number during their initial screening visit. Second, during the baseline consent process, participants are asked if they are willing to be contacted by telephone for appointment reminders, as well as follow-ups for missed appointments. They are asked if they are willing to be visited at their primary residence if they miss a study visit, as well as any alternative residences. After participants have consented to participate in the study, a baseline tracking form is completed capturing the couple’s contact and location details, to the extent they have consented. Participants are asked to provide updated contact information at each follow-up visit. If a couple breaks up during the study, each partner is invited to complete a break-up assessment over the telephone. The assessment documents when the couple broke up, if they tested for HIV as an individual or couple before the break-up, and whether or not participation in the intervention contributed to the break-up. If the participant reports being tested between their last visit and the break-up, they are asked to complete a brief testing questionnaire about whether the intervention contributed to their decision to test. After completing the break-up assessment, the couple is discontinued from the study, and study staff will no longer contact them. All participants are provided with referrals to local counselling/support centers.

#### Primary outcome: couples-based voluntary counseling and testing for HIV

To assess whether our primary outcome of HIV testing has been accomplished, we provide CHCT for study participants. If participants report being tested at an alternate site we will request that they re-test at our facilities to confirm the primary outcome. The testing counselor (a female from the study community) acts separately from the intervention and assessment staff and has no other interactions with participants.

CHCT is conducted in fixed spots in the community using mobile caravans. The CHCT walk-in service is provided once a week in each fixed spot and two days a week are reserved for appointments. The couples make appointments by contacting the study staff by text message or telephone call. The walk-in service is also available to couples on the day of randomization (the first group session); couples are allowed to take up the service immediately after randomization irrespective of their study group allocation. This ensures equal exposure and access to testing for both groups, beginning at the time of randomization.

The Centers for Disease Control and Prevention (CDC) model of HIV counselling and testing to couples was adopted [61]. Same-day rapid testing is conducted with strict confidentiality measures, and includes the following provided by a trained CHCT counselor: pre-test counselling, testing, results interpretation, post-test counselling, and referrals to both members of a couple. Pre-test counselling session includes exploring the couple’s life stage and reason for seeking CHCT, HIV risk concerns are also discussed, and possible test results are discussed intensively. Immediately after testing, couples are provided with their results and the CDC procedures for providing concordant or discordant results are followed. Post-test counselling includes prevention, care, and support discussions, and information on community resources. All couples who have come for CHCT or for information about CHCT are given an information pack regarding resources for care and treatment facilities and other important services available in the community and surrounding areas.

#### Quality assurance/quality control

Prior to trial initiation, all staff received training in study protocol and procedures. Training was specific for each team, with some general training for all staff with regard to ethics, confidentiality, and screening for coercion for participation by partners. The intervention team received specific training in providing relationship skills to couples. Training in conducting CHCT was given to the testing counselor in line with CDC and South African testing guidelines. Extensive practice sessions were conducted and observation by the Project Director and site principal investigator (PI) ensured competency for all staff prior to initiation of trial activities.

Quality assurance (QA) is integral to all activities, and is divided into three components: recruitment, surveys, and CHCT and intervention. First, a directly observed model utilizing scoring sheets is used for all three components and employees are provided with feedback immediately after. This directly-observed QA is conducted by a field supervisor in the field routinely for a sample of all couples undergoing each activity. Second, a laboratory-based model is used for QA of CHCT. All HIV testing practitioners are required to perform proficiency panels in field conditions using rapid test kits with blinded sera quarterly. The compulsory proficiency score is 100%; in cases where a score is less than 100% retraining will be offered. An HSRC site senior nurse supervises the process of panel proficiency testing. Finally, the intervention QA is divided into two parts: 1) a directly observed system with scoring sheet; and 2) tape recordings of couples’ counselling sessions. Group session one and group session two QAs are sampled for 5% of total group sessions for all facilitators. The intervention supervisor directly observes facilitation during group sessions and provides scores and feedback to facilitators. Couple counselling sessions are digitally recorded, with participants’ consent. Counsellors listen to each other’s audio files and provide feedback to each other under the guidance of their supervisor. Any staff member that scores in a manner that indicates under-performance is provided additional training and re-evaluated.

#### Data management

For quantitative surveys, the data are collected in the field on mobile phones, and uploaded to a central database. This method has been used in other large-scale studies (over 13,000 participants in total) being conducted at the study site.

#### Data analysis

##### Primary outcome: CHCT

There are two primary outcomes for our trial: 1) participation in CHCT; and 2) sexual risk behavior. We hypothesize that:

1) The proportion of participants who participate in CHCT by 9-month follow-up will be higher in the intervention group than in the control group.

2) The time to participate in CHCT as a couple will be shorter in the intervention group relative to the control group.

Our initial analyses to address these hypotheses will follow an intention-to-treat (ITT) approach and will be unadjusted. To evaluate hypothesis 1, a chi-square test will be used to compare the proportion of couples that participate in CHCT in the intervention group with the proportion of couples in the control group who participate in CHCT. To evaluate hypothesis 2, we will construct Kaplan–Meier curves to display the two groups’ survival functions and use a log-rank test to compare the two groups’ survival times. Both groups are getting equal exposure and access to testing, beginning from the participation in the first group. Couples will be the unit of analysis for hypotheses 1 and 2. The analysis for hypothesis 1 does not account for when the testing actually occurred, but only examines differences between the intervention and control group at the time of final 9-month follow-up. The analysis for hypothesis 2 will allow us to conduct more nuanced comparisons as to the timing of testing, for example, subsequent to the baseline survey, first intervention group, or follow-up interviews. Thus, we will have our primary comparison of intervention versus comparison group HIV testing within the entire study period at the final follow-up as our primary outcome, and other analyses will further illuminate specific patterns of testing over the course of the study due to intervention participation or exposure. For example, it may be possible that the couples in the intervention condition may exhibit an average longer time to testing when compared to those in the control condition. We will be able to investigate any group differences in timing of testing via our proposed time-to-event analyses.

#### Primary outcome: sexual behavior

In addition to CHCT, our intervention is designed to reduce sexual risk behavior for HIV. We hypothesize that:

1) During follow-up, intervention participants will report fewer acts of unprotected sexual intercourse with: a) primary sex partners; and b) outside sex partners, relative to participants in the control group.

2) During follow-up, intervention participants will report fewer concurrent outside (that is, secondary) sex partners relative to participants in the control group.

We will test longitudinal hypotheses using random effects models to examine changes in participant trajectories in sexual risk outcomes. These longitudinal analyses will treat the individual as the unit of analysis and will include an additional random intercept to account for within-couple response correlations. Initial analyses of sexual risk behavior will follow an ITT approach and include intervention group assignment, time of measurement, and an intervention group-by-time interaction term.

It is critical to optimize the accurate reporting of self-reported sexual risk behavior given its role as a primary outcome of our trial. To account for any possible bias, we have taken the following precautions: 1) we are using previously validated instruments; and 2) the assessment and intervention activities are conducted by different staff members. Thus, we do not expect any differential effects of social desirability reporting across arms of the study.

#### Power analysis

With projected attrition of 20% due to break-ups and 10% due to other causes, we require 350 couples to be randomized in order to achieve the sample size target at 9 months of 245 couples at the final 9-month follow-up. As stated, our primary outcome is determined for only the final follow-up time point, and not for any other group differences at other intervention time points (for example, between first and second group session). The actual prevalence of couple HIV testing is unknown. Thus, for the proposed logistic regression analyses we computed the minimum detectable odds ratio assuming a low prevalence of couple HIV testing (10%) and a medium prevalence of couple HIV testing (30%). For these scenarios, the minimum detectable odds ratios were 2.85 and 2.18, respectively. This was calculated for 80% power. Given the resource-intensiveness of this study intervention, we believe a substantial odds ratio is needed to merit rollout of such an intervention on a public health scale. For time to couple HIV testing survival analyses, under the same assumptions, the minimum detectable hazard ratios are 2.70 and 1.77.

For our sexual behavior outcomes, the minimum detectable R^2^ values fall below 1% for this continuous variable, which is considered a ‘small’ effect size according to Cohen’s [62] criteria for the R^2^ statistic. We based our calculations on data which reported a base rate of 2.35 unprotected sex acts per 3 months (from the study by Genberg and colleagues [6]). We have 80% power to detect a reduction in the mean number of unprotected sex acts from a base rate of 2.35 to 2.04 or 2.12 depending upon the level of within-participant correlation of reported sex acts over time.

## Discussion

The Uthando Lwethu intervention is the first couples-based intervention to have CHCT as its outcome, an approach that has been advocated but not yet implemented widely [16]. The intervention is a theory-based relationship-focused intervention that emphasizes the improvement of positive relationship dynamics and communication skills as they are hypothesized to be the mechanisms by which couples choose to engage in CHCT and reduce sexual risk for HIV. Motivations to participate in couples-based testing for HIV are relationship-focused [18]. Partners want to establish trust, demonstrate commitment to each other, and know their HIV status prior to embarking on future decisions such as having children together, or getting married [18]. Prior attempts at couples-based approaches to HIV prevention have more often utilized individually-focused theoretical orientations, thereby neglecting the influence that partners play in decisions around HIV, both its prevention and potential transmission [9,63].

Improving rates of CHCT is a high leverage outcome which has the potential to improve rates of testing, disclosure, sexual risk reduction, and treatment initiation for heterosexual couples in a rural area of South Africa which is highly impacted by HIV. While CHCT has been shown to be efficacious for sexual risk reduction, uptake has been low in South Africa. Our strategies are aiming to address factors that may have contributed to the historically low uptake of CHCT. For example, we are utilizing a community-based approach, which is likely to increase our access to and participation of men; we are taking our services to where men are, as opposed to prior work with couples that has been conducted mostly in the context of clinic-based environments (for example, PMTCT interventions). Other innovations include the use of mobile caravans for provision of counseling services, intervention activities and CHCT, and the use of mobile phones for data collection.

Uthando Lwethu integrates relationship-focused education and skills training into an HIV-focused couples-based intervention. In addition, it incorporates relationship education approaches [44,60] and interdependence theory [40] to increase positive relationship dynamics. By specifically targeting relationship commitment, intimacy, and satisfaction we are directly intervening in the context of HIV risk in South Africa, that is, primary relationships. Thus, our intervention has the aim of both increasing CHCT (thus increasing mutual knowledge of serostatus), reducing sexual risk behavior for HIV with both primary and secondary partners (perhaps in the absence of knowledge of serostatus), and improving our understanding of how relationship issues influence these behavioral choices. This represents an attempt to move away from individual-level approaches, to a more integrated approach for HIV prevention.

Couples-based interventions for HIV, including those that target testing and sexual risk reduction have been identified as a high priority [11,38,63]. CHCT necessitates the testing and mutual disclosure of both partners, conditions that are essential for improving subsequent outcomes such as sexual risk reduction and improving treatment outcomes. Although interventions aimed towards improving rates of testing for male partners of antenatal clinic attendees have had similar goals, they have reported mixed results [64,65].

We will learn from our intervention whether conducting a couples-based, relationship-focused intervention is feasible in a context which presents unique challenges for couples, for example, low rates of marriage, high rates of mobility. Should the intervention demonstrate efficacy, next steps could include a rollout of this public health intervention, as well as adapting and testing our approach for couples who are experiencing current intimate partner violence, substance use, or pregnant women.

Heterosexual couples in this context are at high risk for HIV infection, and engaging in CHCT could garner positive outcomes for primary and secondary prevention as well as treatment outcomes [11] (for example, earlier ART initiation, reduced HIV transmission risk stemming from early ART, access to PrEP, and so on), which can significantly impact public health outcomes in this community. Mutual disclosure of serostatus can also improve social support and relationship quality, in addition to facilitating successful sexual risk reduction. The results of our ongoing clinical trial will provide much needed information regarding whether a relationship-focused approach is effective in increasing rates of participation in CHCT. Choosing to engage in CHCT represents a crucial decision for couples with significant implications for their psychological and physical welfare. Our results will indicate whether this decision can be facilitated by providing couples with skills to improve their communication not only about their sexual behavior and decisions, but about the relationship context in which these decisions take place.

## Trial status

Recruitment for the trial began in March 2012 and will continue through 2014. As of June 2013, we have enrolled 155 couples.

## Abbreviations

ART: Antiretroviral therapy; BMT: Behavioral marital therapy; CAB: Community Advisory Board; CDC: Centers for Disease Control and Prevention; CHCT: Couples-based HIV counseling and testing; DSMB: Data Safety and Monitoring Board; ITT: Intention-to-treat; PI: Principal investigator; PMTCT: Prevention of mother-to-child transmission; PReP: Pre-exposure prophylaxis; PREP: Prevention and Relationship Enhancement Program; QA: Quality assurance; RCT: Randomized controlled trial; STI: Sexually transmitted infection; TB: Tuberculosis; WHO: World Health Organization.

## Competing interests

The authors declare that they have no competing interests.

## Authors’ contributions

LAD contributed to study conception, design, drafting, and critically revising the manuscript. HVR, VH, MOJ, KF, and NM contributed to study conception, design, and critically revising the manuscript. TN contributed to the acquisition of data and critically revising the manuscript. All authors read and approved the final manuscript.
